# Multiple Perspectives on Disaster Preparedness in Long-Term Care: From Heat to Hospice

**DOI:** 10.1093/geroni/igab046.1100

**Published:** 2021-12-17

**Authors:** David Dosa, Ross Andel, Lisa Brown

## Abstract

Preparedness of residents in long-term care (LTC) exposed to disasters continues to warrant concern. Prior work by our research team highlights explicit evidence of the profound vulnerability of Florida nursing home (NH) residents exposed to Hurricane Irma in 2017. This research adds to our knowledge of the profound effect of disasters on long term care residents. This symposium will utilize mixed methodologies to discuss the varied effects of Hurricane Irma on vulnerable older adults residing in Florida NHs and Assisted Living communities (ALCs). Using a novel methodology for identifying a cohort of ALC residents, the first presentation will present the morbidity and mortality effects of Hurricane Irma on Florida ALC residents and identify high risk groups by health condition. The second presentation will document the effect of Hurricane Irma on NH Residents previously enrolled in Hospice and expound on the effect of the disaster on hospice enrollment after the storm. The third presentation will present qualitative results of interviews with ALC administrators highlighting the effect of the storm on both large and small ( <25 beds) facilities. The fourth presentation will address the issue of heat exposure in the days after Hurricane Irma and consider the preventative effect of generators on morbidity and mortality. Finally, a fifth presentation will examine NH staffing level variation in the days leading to the hurricane. To conclude, this symposium offers a multi-faceted view of a disaster’s effects on LTC residents across Florida, including novel data from the NH environment and lesser-examined ALCs.

